# High fractional excretion of glycation adducts is associated with subsequent early decline in renal function in type 1 diabetes

**DOI:** 10.1038/s41598-020-69350-y

**Published:** 2020-07-29

**Authors:** Bruce A. Perkins, Naila Rabbani, Andrew Weston, Antonysunil Adaikalakoteswari, Justin A. Lee, Leif E. Lovblom, Nancy Cardinez, Paul J. Thornalley

**Affiliations:** 1 Division of Endocrinology and Metabolism, Department of Medicine, University of Toronto, Mount Sinai Hospital, Toronto, ON Canada; 2 Department of Basic Medical Science, College of Medicine, QU Health, Qatar University, P.O. Box 2713, Doha, Qatar; 3 Clinical Sciences Research Institute, Warwick Medical School, University of Warwick, Coventry, UK; 4 Present Address: University College London School of Pharmacy, 29-39 Brunswick Square, London, WC1N 1AX UK; 5 Present Address: School of Science and Technology, Nottingham Trent University, Clifton Lane, Nottingham, NG11 8NS UK; 6 Diabetes Research Center, Qatar Biomedical Research Institute, Hamad Bin Khalifa University, Qatar Foundation, P.O. Box 34110, Doha, Qatar

**Keywords:** Biochemistry, Biomarkers

## Abstract

Increased protein glycation, oxidation and nitration is linked to the development of diabetic nephropathy. We reported levels of serum protein glycation, oxidation and nitration and related hydrolysis products, glycation, oxidation and nitration free adducts in patients with type 1 diabetes (T1DM) during onset of microalbuminuria (MA) from the First Joslin Kidney Study, a prospective case–control study of patients with T1DM with and without early decline in GFR. Herein we report urinary excretion of the latter analytes and related fractional excretion values, exploring the link to MA and early decline in GFR. We recruited patients with T1DM and normoalbuminuria (NA) (n = 30) or new onset MA with and without early GFR decline (n = 22 and 33, respectively) for this study. We determined urinary protein glycation, oxidation and nitration free adducts by stable isotopic dilution analysis liquid chromatography-tandem mass spectrometry (LC–MS/MS) and deduced fractional excretion using reported plasma levels and urinary and plasma creatinine estimates. We found urinary excretion of pentosidine was increased *ca.* twofold in patients with MA, compared to normoalbuminuria (0.0442 vs 0.0103 nmol/mg creatinine, *P* < 0.0001), and increased *ca.* threefold in patients with early decline in GFR, compared to patients with stable GFR (0.0561 vs 0.0176 nmol/mg creatinine, *P* < 0.01). Urinary excretion of all other analytes was unchanged between the study groups. Remarkably, fractional excretions of 6 lysine and arginine-derived glycation free adducts were higher in patients with early decline in GFR, compared to those with stable GFR. Impaired tubular reuptake of glycation free adducts by lysine and arginine transporter proteins in patients with early GFR decline is likely involved. We conclude that higher fractional excretions of glycation adducts are potential biomarkers for early GFR decline in T1DM and MA. Measurement of these analytes could provide the basis for identifying patients at risk of early decline in renal function to target and intensify renoprotective treatment.

Diabetic nephropathy is the leading cause of end-stage renal disease (ESRD) and severely decreases likelihood of long-term survival of patients with diabetes^1^. It has high occurrence linked to a high global prevalence of diabetes, 5% type 1 diabetes mellitus (T1DM) and 95% type 2 diabetes mellitus (T2DM), with *ca.* 40% of patients with diabetes developing diabetic nephropathy^2,3^. Approximately half of patients with diabetic nephropathy progress to diabetic kidney disease and ESRD with increased risk of potentially fatal cardiovascular disease. Current treatment guidelines address modifiable risk factors through intensification of control of glycemic status, blood pressure and lipids^4^. This targets prevention of development of overt proteinuria and decline of GFR to ESRD. The effectiveness of current treatments achieves only slowing of decline in GFR. There is also high on-treatment mortality, mostly from fatal cardiovascular disease^5^. Recent advances have been made through benefits found from treatment with inhibitors of sodium–glucose cotransporter 2 (SGLT2)^6^. Early diagnosis of patients at high risk of rapid decline of renal function and intensive management with SGLT2 inhibitors and other therapy may improve treatment outcomes. There is an urgent need for biomarkers capable of predicting early GFR decline for targeted intensive therapy and to improve understanding of the mechanisms involved in renal injury to guide development of more effective renoprotective agents.

Multiple mechanisms of metabolic dysfunction have been proposed to explain the link of diabetic nephropathy to glycemic control. One is the increased formation and accumulation of advanced glycation endproducts (AGEs). AGEs are formed by the degradation of fructosamine adducts, such as N_ε_-fructosyl-lysine (FL), of glucose-modified proteins and by the direct modification of proteins by reactive dicarbonyl metabolites, such as methylglyoxal. Examples of major AGEs quantitatively found in the clinical setting are: hydroimidazolone, MG-H1—formed by modification of arginine residues with methylglyoxal; and N_ε_-carboxymethyl-lysine (CML) – formed mainly by the oxidative degradation of FL residues. There is also a minor, trace level AGE protein crosslink with intense fluorescence called pentosidine, formed by the reaction of pentose metabolites with spatially close arginine and lysine residues. Protein glycation adducts exist in mainly two forms: glycation adduct residues of proteins – sometimes called “protein-bound” glycation adducts, and glycation free adducts or glycated amino acids. Glycation free adducts are formed mostly from proteolysis of endogenous glycated proteins with also a contribution from digestion of glycated proteins in food – as recently reviewed^7^. AGEs have long been considered as risk predictors of diabetic nephropathy and other microvascular complications of diabetes^8^. Analysis of skin collagen in patients with T1DM showed that a combination of AGEs and the FL-linked analyte, furosine, was linked to risk of progression of diabetic nephropathy^9^. A blood and/or urine-based biomarker would provide more convenient clinical sampling for risk prediction of nephropathy progression. Exploring this, plasma protein content of CML was examined and found to be not linked to the risk of developing diabetic nephropathy^10^. In patients with T1DM and normoalbuminuria (NA), plasma MG-H1 free adduct concentration was an independent risk predictor for increased thickening of glomerular basement membrane measured in renal biopsies, linked to early stage development of diabetes nephropathy^11^. Oxidative stress has also been implicated in the development of diabetic nephropathy through increased oxidative damage to renal proteins, including formation of the protein nitration adduct, 3-nitrotyrosine (3-NT)^12^. Protein glycation and oxidative damage of multiple chemically-defined types may be quantified robustly and concurrently by stable isotopic dilution analysis liquid chromatography-tandem mass spectrometry (LC–MS/MS)^13^. In a previous study of patients with T1DM, we found changes in serum protein glycation adducts, related serum free adducts and also protein oxidation and nitration adduct levels between patients with NA and new onset microalbuminuria (MA) but no difference between patients with MA with later stable or declining renal function^14^.

Glycated, oxidized and nitrated amino acids have molecular mass < 500 Da. They pass readily through the glomerular filter and are excreted in urine^15^. Plasma levels vary from 1 – 500 nM and urinary concentrations from 10 nM to 100 µM with chemical half-lives ranging from 2 weeks (hydroimidazolone MG-H1) to many years (CML) under physiological conditions^13,15,16^. The low level of glycation, oxidation and nitration free adducts is due to the low in situ rate of protein glycation, oxidation and nitration. Protein glycation by reactive dicarbonyls is suppressed by enzymes such as glyoxalase 1 and aldoketo reductases^17^ and protein oxidation and nitration is suppressed by antioxidants^13^. Levels of FL are suppressed by repair of this glycation adduct catalyzed by fructosamine-3-phosphokinase^18^. They have different and characteristic renal clearances due to differential reuptake in renal tubules^16^. We hypothesised that renal handling of glycation, oxidation and nitration free adducts, as judged by fractional excretion, may be sensitive to early decline in renal function and thereby a biomarker of subsequent decline in renal function. In this study we investigated urinary excretion and fractional excretion of glycation, oxidation and nitration free adducts as indicators of their total exposure and relative renal clearance, respectively. The outcome suggests fractional excretion of 6 lysine and arginine-derived glycation adducts are potential biomarkers for early GFR decline in T1DM and MA.

## Results

### Clinical characteristics of diabetic patients in this study

Eighty-five patients with T1DM were recruited for this study. The baseline clinical characteristics are given in Table 1. Thirty patients had NA and 55 had MA. Of the 55 patients with MA, 22 had early decline in GFR, defined as a decline in GFR > 3.2% per year, and the remaining 33 had stable GFR. All 30 patients with NA had stable GFR. In statistical comparisons, comparing patients with MA (with and without early decline in GFR combined) to patients with NA, patients with MA had a younger age, a higher percentage of current smokers, higher glycated hemoglobin A1c, serum cystatin C and, by definition, urinary albumin excretion. Comparing patients with MA and early GFR decline to patients with stable GFR with and without MA combined, patients with early decline in GFR had a higher percentage of current smokers, higher glycated hemoglobin A1c, plasma total cholesterol and triglycerides, urinary albumin excretion and serum cystatin C, and lower GFR_CYSTATIN C_.

**Table 1:** Baseline clinical characteristics of patients in this study.

Characteristic	Normoalbuminuria (n = 30)	Microalbuminuria (n = 55)		*P *value NA vs. MA	*P *value NA and stable GFR vs. early GFR decline
		Stable GFR (n = 33)	Early GFR Decline (n = 22)		
Gender (female, %)	18 (60%)	16 (48%)	13 (59%)		
Age (years)	41.9 ± 6.2	36.3 ± 8.8	37.8 ± 8.0	< 0.01	
Duration of diabetes (years)	26.3 ± 8.3	23.0 ± 8.2	23.6 ± 8.3		
Current smoking (%)	1 (3%)	13 (39%)	11 (50%)	< 0.001	< 0.001
Systolic blood pressure (mmHg)	118.4 ± 11.0	121.6 ± 14.9	128.3 ± 20.1		
Diastolic blood pressure (mmHg)	73.4 ± 6.2	76.0 ± 10.5	76.6 ± 8.7		
Glycated hemoglobin A1c (%)*	7.81 ± 1.25	8.73 ± 1.10	9.92 ± 1.48	< 0.001	< 0.001
Total cholesterol (mg/dL)*	190 ± 34	187 ± 40	223 ± 39		< 0.001
Triglycerides (mg/dL)*	107 ± 97	97 ± 56	157 ± 117		0.01
Urinary albumin excretion rate (µg/min)*					
2-year baseline interval	11.9 [9.7–12.3]	45.1 [32.4–58.2]	47.1 [32.8–55.1]	< 0.001	
At time of adduct measurement	6.4 [3.4–13.6]	74.3 [35.2–66.8]	59.8 [36.5–85.5]	< 0.001	< 0.001
Serum cystatin C (mg/L)	0.72 ± 0.08	0.77 ± 0.13	0.85 ± 0.27	< 0.05	< 0.01
GFR_CYSTATIN C_ (ml/min/1.73m^2^)	117.1 ± 13.6	111.9 ± 18.3	104.6 ± 25.8		< 0.05

Data are mean ± SD or median [interquartile range]. All participants in the normoalbuminuria group had stable GFR. Abbreviations: NA, normoalbuminuria group; MA, microalbuminuria group. *These values represent the mean values for all measurements taken during the two-year baseline interval used for classification of new onset microalbuminuria.

### Urinary excretion of protein glycation, oxidation and nitration free adducts

**Figure 1:**
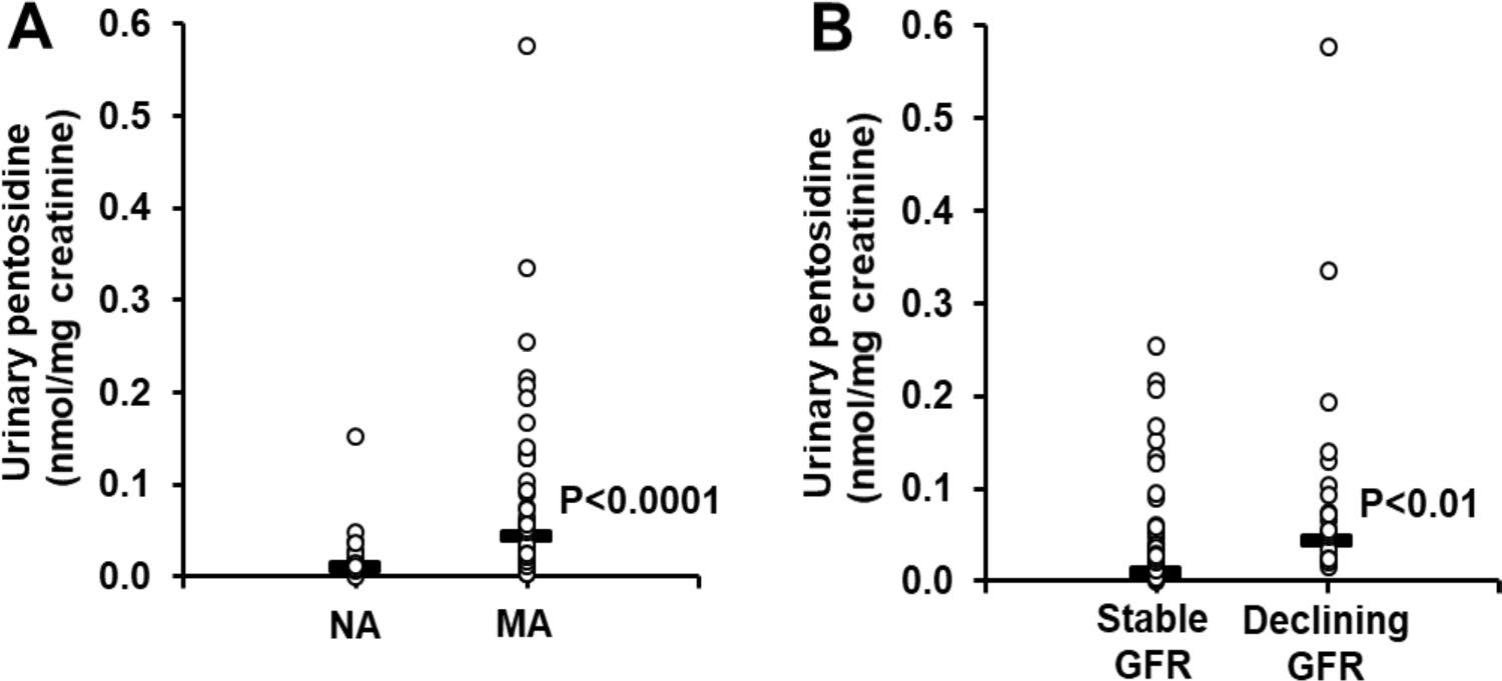
Urinary excretion of pentosidine in cases of new onset microalbuminuria with and without Early GFR decline compared to normoalbuminuria controls. (**a**) Increased urinary excretion of pentosidine in patients with MA, with respect to patients with NA. (**b**) Increased urinary excretion of pentosidine in patients with early decline in GFR, with respect to patients with stable renal function. Data distributions are shown with horizontal bars indicating median values. Statistical analysis: Mann–Whitney U test.

We present data of urinary excretion of protein glycation, oxidation and nitration free adducts in the patient study groups at baseline—Table 2. In the normoalbuminuria patient study group, the urinary excretion of glycation adducts was in the approximate order: MG-H1 > FL > CML > CMA ≈3DG-H≈CEL > MOLD > GOLD > pentosidine. The urinary excretion of oxidation and nitration adducts was in the approximate order: MetSO > NFK > DT > 3-NT. The urinary excretion of protein glycation, oxidation and nitration free adducts between patient study groups with and without MA and early GFR decline was remarkably similar. Only urinary excretion of pentosidine free adduct was significant between study groups (*P* < 0.001, *Kruskal–Wallis* test). Nevertheless, patients with MA with and without early GFR decline had higher urinary excretion of pentosidine, compared to patients with NA (0.0442 vs 0.0103 nmol/mg creatinine, *P* < 0.001); and patients with early decline in GFR had higher urinary excretion of pentosidine, compared to patients with stable renal function with and without MA (0.0561 vs 0.0176, *P* < 0.01)—Fig. 1.

**Table 2:** Urinary excretion of glycation, oxidation and nitration free adducts (nmol/mg creatinine).

Urinary free adduct	Normoalbuminuria (n = 30)	Microalbuminuria (n = 55)		*P *value NA vs. MA*	*P *value NA and stable GFR vs. early GFR decline*
		Stable GFR (n = 33)	Early GFR decline (n = 22)		
FL	32.0 [22.3–42.3]	37.5 [13.5–71.7]	60.6 [30.9–99.4]		
CML	3.30 [2.33–4.70]	2.63 [1.11–5.94]	4.04 [2.87–8.45]		
CEL	1.22 [0.90–1.61]	1.06 [0.42–2.53]	1.80 [1.18–3.29]		
G-H1	1.92 [1.29–2.39]	1.42 [0.42–2.99]	1.78 [1.33–2.64]		
MG-H1	61.9 [30.4–92.3]	42.7 [13.0–93.1]	51.9 [29.2–155.3]		
3DG-H	1.34 [1.04–1.94]	0.80 [0.39–1.65]	1.42 [0.74–1.95]		
CMA	1.61 [1.25–2.24]	1.70 [0.66–3.66]	2.04 [1.36–4.03]		
GOLD	0.0126 [0.0092–0.0207]	0.0189 [0.0057–0.0243]	0.0204 [0.0101–0.0249]		
MOLD	0.069 [0.050–0.107]	0.097 [0.065–0.139]	0.109 [0.076–0.158]		
Pentosidine	0.0102 [0.0064–0.0170]	0.0313 [0.0144–0.0905]	0.0561 [0.0273–0.1027]	< 0.0001	< 0.01
MetSO	1.12 [0.56–1.93]	0.83 [0.44–1.64]	0.60 [0.30–1.20]		
DT	0.0529 [0.0404–0.0639]	0.0325 [0.0175–0.1036]	0.0547 [0.0392–0.0950]		
NFK	0.285 [0.205–0.352]	0.138 [0.042–0.320]	0.189 [0.077–0.282]		
3-NT	0.0094 [0.0041–0.0169]	0.0136 [0.0045–0.0301]	0.0117 [0.0050–0.0369]		

Data are median [interquartile range]. *A Bonferroni correction of 14 was applied. Data from 3 patients are missing (2 from MA with stable renal function and one from MA with early decline in renal function).

### Fractional excretion of protein glycation, oxidation and nitration free adducts

**Figure 2:**
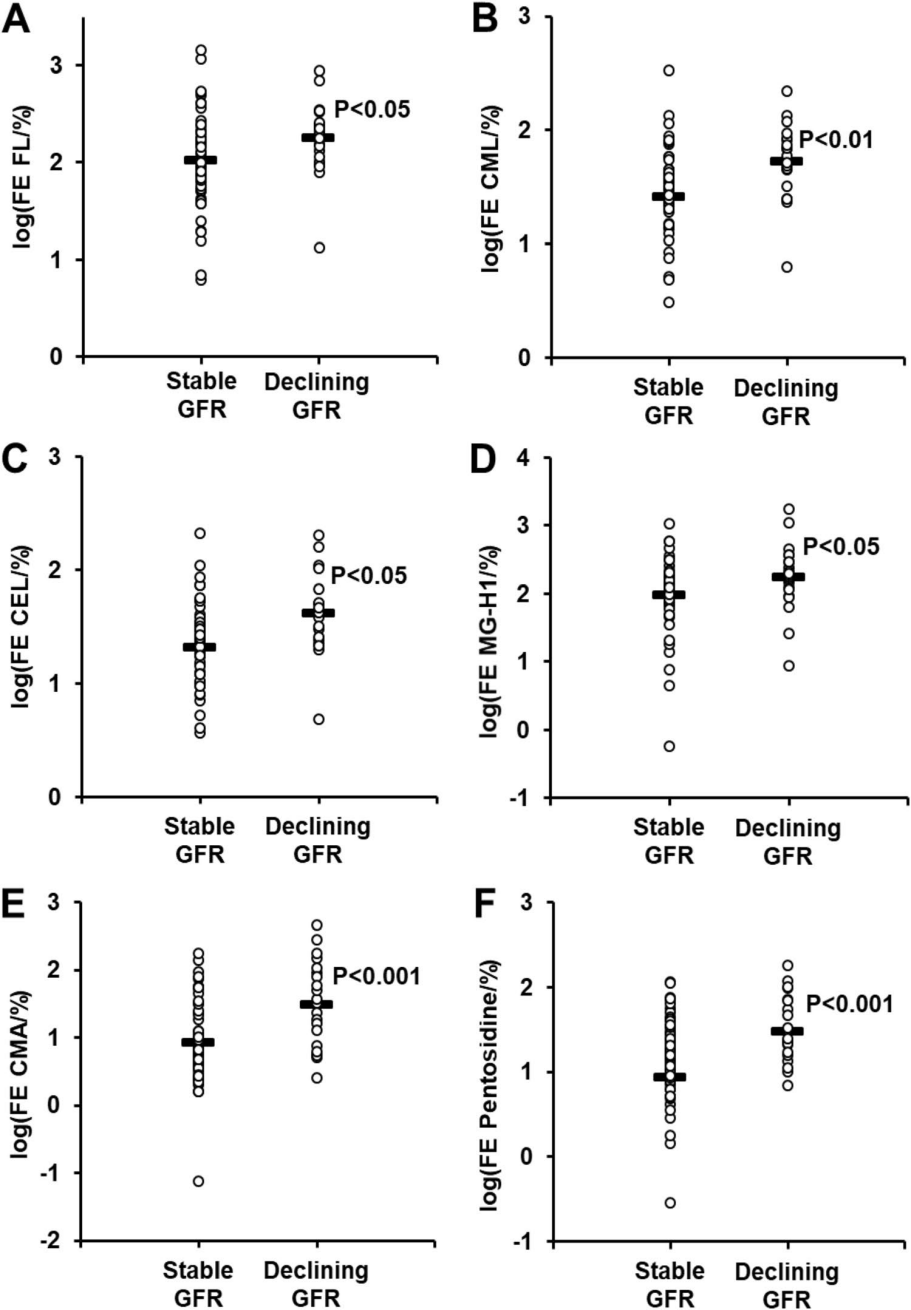
Fractional excretion of glycation free adducts in cases of new onset microalbuminuria with and without early GFR decline compared to normoalbuminuria controls. Increased fractional excretion (FE) of glycation free adducts in patients with early decline in GFR, with respect to patients with stable renal function. Data are log(FE_glycation adduct_/%). Panel key: (**a**) FL. (**b**) CML. (**c**) CEL. (**d**) MG-H1. (**e**) CMA. (**f**) Pentosidine. Data distributions with horizontal bars indicating log(mean) values. Statistical analysis: Student’s t-test of log_10_ transformed values.

Fractional excretion of protein glycation, oxidation and nitration free adducts were deduced from the concentrations of these analytes in serum, described previously^14^, urinary fluxes presented herein and creatinine concentrations in serum and urine—Table 3. In patients with normoalbuminuria there was a wide range of fractional excretion values of protein glycation, oxidation and nitration free adducts, ranging from 0.8% for MetSO to 100% for FL. Patients with MA with and without early function decline had decreased fractional excretion of G-H1 (14.5 vs 30.7%, *P* < 0.01) and increased fractional excretion of CMA (22.4 vs 4.3%, *P* < 0.001) and pentosidine (97.1 vs 8.8%, *P* < 0.001), compared to patients with NA. Patients with early decline in GFR had higher fractional excretion of 6 lysine and arginine-derived glycation adducts, compared to patients with stable function. Glycation adducts with higher fractional excretion were: FL, CML, CEL and MG-H1—all increased *ca*. twofold; and CMA and pentosidine, increased *ca.* sixfold and fivefold, respectively—Table 2. Data distributions of log transformed values are given in Fig. 2. There was no similar increase in fractional excretion of unglycated lysine and arginine in early GFR decline: lysine, stable GFR 0.41 [0.27–0.75]%, early decline in GFR 0.40 [0.28–0.67]%; and arginine, stable GFR 0.51 [0.21–0.83], early decline in GFR 0.39 [0.22–0.65].

**Table 3:** Fractional Excretion of Protein Glycation, Oxidation and Nitration Free Adducts (%).

Free adduct	Normoalbuminuria (n = 30)	Microalbuminuria (n = 55)	*P *value (NA vs MA)	NA and stable GFR (n = 63)	Early GFR decline (n = 22)	*P *value stable vs early GFR decline
FL	100 [54–160]	153 [79–247]		100 [58–204]	204 [114–257]	< 0.05
CML	30.4 [25.0–39.4]	43.6 [22.1–71.9]		30.6 [21.7–43.5]	52.3 [44.8–81.9]	< 0.01*
CEL	26.4 [16.0–33.7]	28.3 [18.1–52.9]		25.1 [12.6–34.4]	42.4 [26.0–52.0]	< 0.05
G-H1	30.7 [15.0–60.0]	14.5 [5.3–30.0]	< 0.01*	19.6 [6.9–39.6]	17.9 [13.4–44.1]	
MG-H1	113 [91 -146]	123 [73–263]		111 [71–156]	182 [115–290]	< 0.05
3DG-H	15.0 [11.6–23.5]	15.8 [8.4–27.9]		14.0 [8.9–22.7]	19.8 [14.1–39.9]	
CMA	4.3 [2.9–5.6]	22.4 [7.2–57.5]	< 0.001*	6.0 [3.9–23.6]	32.2 [7.6–89.6]	< 0.001*
Pentosidine	8.8 [5.0–20.3]	97.1 [38.2–141.1]	< 0.001*	20.9 [7.3–94.9]	109.1 [74.6–143.8]	< 0.001*
ΣERFD predictors	285 [223–387]	537 [293–867]	< 0.05*	337 [217–526]	785 [499–991]	< 0.001*
MetSO	0.8 [0.3–1.6]	0.6 [0.4–1.3]		0.7 [0.3–1.5]	0.6 [0.4–1.4]	
DT	26.7 [20.9–43.8]	27.8 [12.5–56.8]		26.6 [15.6–50.6]	29.4 [19.0–49.7]	
NFK	8.8 [5.7–14.3]	6.6 [2.6–10.7]		7.7 [3.5–11.0]	9.2 [3.7–12.1]	
3-NT	10.5 [4.2–29.4]	13.4 [5.7–26.6]		13.3 [4.6–26.5]	11.8 [7.7–30.5]	

Data are median [interquartile range]. Statistical analysis was performed using the Student’s t-test of log-transformed values. *Significance of difference remained significant after a Bonferroni correction of 12 was applied. Data from 3 patients are missing (2 from MA with stable renal function and one from MA with early decline in renal function). ΣERFD predictors is the sum of FE values for FL, CML, CEL, MG-G1, CMA and pentosidine.

### Correlation analysis

We explored correlations of clinical parameters and the urinary excretion and fractional excretion of protein glycation, oxidation and nitration free adducts for all study group patients combined (applying Bonferroni corrections of 14 and 12 respectively; *P* < 0.05). For urinary excretion of free adducts, we found that total plasma cholesterol was positively correlated with urinary excretion of FL (r = 0.32), CML (r = 0.34), and CEL (r = 0.31). Patient age was positively correlated with urinary excretion of NFK (r = 0.33). Diabetes duration was positively correlated with urinary excretion of NFK (r = 0.36). In addition, female sex was associated with higher urinary excretion of GOLD (0.0107 versus 0.0193 nmol/mg creatinine). For fractional excretion, there were positive correlations of glycated hemoglobin A1c with fractional excretions of CML (r = 0.42) and CEL (r = 0.36), and total plasma cholesterol with fractional excretion of CML (r = 0.34). Diabetes duration was positively correlated with fractional excretion of NFK (r = 0.32). Blood pressure and current smoking status were not associated with any of the adducts.

We also studied correlation of the flux of urinary free adducts at baseline with GFR _CYSTATIN C_, urinary albumin excretion rate (AER) and slope of future decline in GFR (%/year). There were no significant correlation of urinary protein glycation, oxidation and nitration adduct with GFR _CYSTATIN C._ For AER, there was a positive correlation with urinary excretion of pentosidine (r = 0.43, *P* < 0.001). We found future rate of decline in GFR correlated negatively with urinary CEL (r =− 0.35, *P* < 0.01) and urinary pentosidine (r =− 0.42, *P* < 0.001). This may suggest that for patients with diabetic nephropathy, future early decline in renal function is linked to increased exposure to the glycating agent, methylglyoxal, and increase pentosephosphate pathway metabolism leading to increased formation of pentosidine. Future rate of decline in GFR correlated negatively with fractional excretion of CML (r =− 0.41, *P* < 0.01), CEL (r =− 0.36, *P* < 0.05), MG-H1 (r =− 0.37, *P* < 0.05), 3DG-H (r =− 0.36, *P* < 0.05), CMA (r =− 0.36, *P* < 0.05) and pentosidine (r =− 0.41, *P* < 0.01).

## Discussion

In this study we present for the first-time evidence that fractional excretions of 6 lysine and arginine-derived glycation adducts are higher in patients with T1DM and early decline in GFR, compared to patients with T1DM and stable renal function with or without MA. This suggests that fractional excretion of these glycation free adducts (glycated amino acids) may be a risk predictor of subsequent rapid decline in renal function. This is of interest to validate in a future study and independent cohort, including in patients with type 2 diabetes mellitus. If validated, the measurement of these analytes is simple and could provide the basis for identifying patients at risk of early decline in renal function to target and intensify renoprotective treatment. Fractional excretion may be further affected by weight, age and gender, although these are generally rather considered linked to urinary flux of creatinine and not to the specific analytes^19,20^.

**Figure 3:**
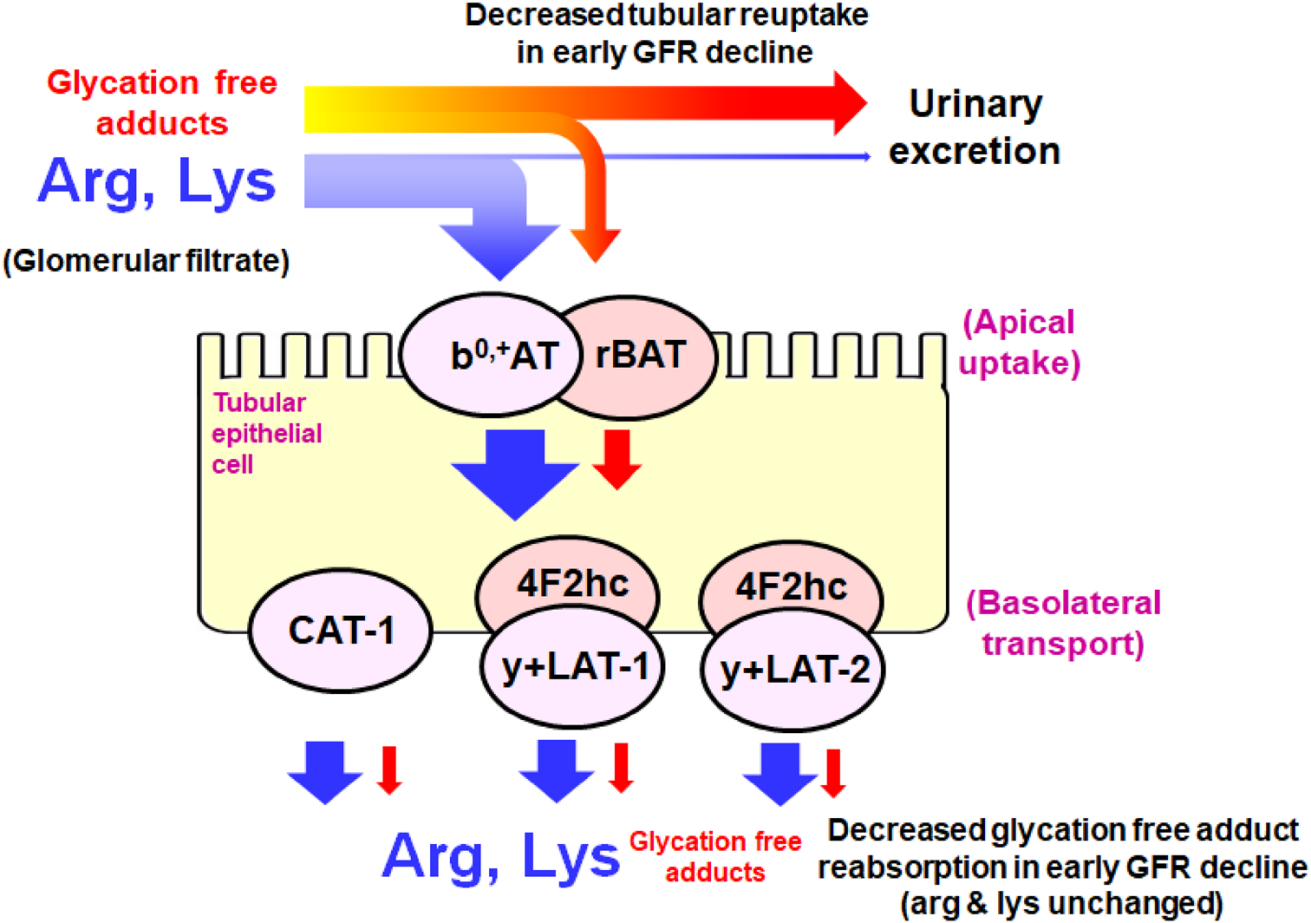
Schematic diagram of amino acid transporters of arginine and lysine uptake in the renal tubular epithelium and engagement with glycation free adducts. See Discussion for description.

Protein glycation, oxidation and nitration are endogenous spontaneous processes occurring in healthy subjects. The in situ rates of protein glycation, oxidation and nitration are increased in patients with T1DM. Relatedly, there are increased levels of protein glycation, oxidation and nitration free adducts in plasma and urine of patients with T1DM, compared to healthy controls^16^. The flux of formation of protein glycation, oxidation and nitration free adducts in patients with T1DM with and without early renal function decline, as judged by the urinary flux of free adducts, was unchanged—except for increase of pentosidine in early renal function decline. The observed higher fractional excretion of glycation free adducts is likely an indicator of decline in renal function and more sensitive to early renal function decline than serum creatinine or cystatin C. As glycation free adducts analytes pass readily through the glomerular filter, this effect is likely due to decreased tubular reuptake of glycation free adducts. Related increased renal clearance of glycation free adducts in stable renal function was found previously in streptozotocin-induced diabetic rats with albuminuria and stable renal function^21^. We also found higher renal clearance of glycation free adducts in patients with T1DM with normal renal function, compared to healthy controls^16^. For FL there may also be lower renal metabolism by fructosamine 3-phosphokinase associated with decreased reuptake by the tubular epithelium^22^. Pentosidine also undergoes tubular reuptake and metabolism in the kidney and this may also be susceptible to decrease in the setting of early decline of GFR^23^. Increased fractional excretion of multiple lysine and arginine-derived glycation adducts was found, suggesting that the effect is not linked to a particular glycation process. Rather, the higher fractional excretions are likely indicative of change in activity of transport proteins mediating reuptake of glycation free adducts in the tubular epithelium. Lysine and arginine-derived glycation free adducts are thought to be taken up and moved across the tubular epithelium by low affinity binding to cation transporter proteins which also take up arginine and lysine, heterodimeric complex b^0,+^AT/rBAT on the apical surface (gene names SLC7A9 and SLC3A1, respectively) and CAT-1 and heterodimeric complexes of y + LAT1, y + LAT2 with 4F2hc on the basolateral surface (gene names SLC7A1, SLC7A7, SLC7A6 and SLC3A2, respectively)^24–28^—Fig. 3. Interestingly, CAT-1 is regulated by glucose and insulin and may suffer periodic variation in patients with T1DM^29^; and in genome-wide association studies genetic polymorphism of SLC7A7, SLC7A7 and SLC7A9 was associated with variation in eGFR and development of chronic kidney disease^30,31^. There was no similar increase in fractional excretion of arginine and lysine. This may be because glycation free adducts have markedly low binding affinity to cation transporters and higher fractional excretions compared to arginine and lysine^24–26^. This weak binding affinity of glycation free adducts is expected to confer greater sensitivity to decline in transporter activity than the corresponding unmodified amino acids.

In some patients, mostly those with early GFR decline, fractional excretion of FL, MG-H1 and pentosidine exceeded 100%; renal clearance of these glycation free adducts was greater than that of creatinine. This is likely mediated by high active tubular secretion and may be an indication of the tubular epithelium under physiological stress related to subsequent decline in GFR^32^. Active tubular secretion is mediated by organic anion transporters (OATs) and organic cation transporters (OCTs) on the basolateral side and multidrug and toxin extrusion proteins (MATEs) on the apical side of the tubular epithelium^32^. These transporters may also accept zwitterionic substrates such as glycation free adducts^33^. Increased flux of glycation free adducts and other ionic substrates through the tubular epithelium may be an early-stage functional change contributing to subsequent decline in renal function.

In contrast, fractional excretion of oxidation free adducts was not associated with MA status and early decline in GFR. Fractional excretion of MetSO was very low (< 1%), as expected for the high renal metabolism of MetSO by MetSO reductases^34^. DT, as a dipeptide-like structure, may have tubular reuptake by peptide transporter protein such as H + –peptide co-transporter PEPT1^24^, and NFK and 3-NT by the tryptophan and tyrosine transporter proteins, respectively—proteins 4F2hc/LAT1 and TAT1 (genes SLC3A2/SLC7A5 and SLC16A10)^35^. These transporter proteins have no known link to chronic kidney disease. No changes of fractional excretion of DT, NFK and 3-NT with MA and early decline in GFR were found herein.

Other findings reported herein were urinary excretion rates of glycation, oxidation and nitration free adducts in patient study groups. There were remarkably few changes in patient study groups except for increased urinary excretion rates of pentosidine. Pentosidine excretion was *ca.* twofold higher in patients with T1DM and MA, compared to patients with T1DM and NA, and also *ca.* threefold higher in patients with early decline in GFR, compared to patients with stable renal function. Pentosidine is formed from pentose metabolite precursors and is a biomarker of pentosephosphate pathway activity^36^. Urinary excretion of pentosidine may reflect increased endogenous formation of pentosidine associated with increased pentosephosphate activity sustaining formation of NADPH cofactor for aldoketo reductases and glutathione reductase to counter dicarbonyl stress and oxidative stress in diabetes^12,37^. This may become more marked in MA and early decline in GFR with increased dicarbonyl stress and oxidative stress associated with accumulation of renal toxins^38^. Decreased renal metabolism of pentosidine may also contribute to the increased excretion of pentosidine.

Fractional excretion of the glycation adduct G-H1 was lower and fractional excretions of CMA and pentosidine were higher in patients with MA with and without early GFR decline, compared to patients with NA. These effects were maintained when comparing patients with stable renal function with and without MA; that is, these changes relate to increased in urinary albumin in the NA to MA range. Impaired tubular reuptake and/or metabolism of CMA and pentosidine may be stimulated in response to increased albumin in the tubular lumen before decline in GFR begins. These glycation analytes may be particularly sensitive to early dysfunction in the glomerular filter and albumin salvage pathway^39^.

Patients with T1DM have increased exposure to glycating agents, glucose, methylglyoxal, glyoxal and 3-deoxyglucosone linked to insulin deficiency, dysregulated glucose metabolism and glycolysis^40–42^. For example, in an earlier study plasma glucose was increased two–threefold and whole blood methylglyoxal concentrations increased five–sixfold in patients with T1DM^40^. Consistent with this, there is increased endogenous glycation of proteins with cell proteolysis leading to the increased formation and excretion of glycation free adducts^21^. Consequently, urinary excretion of FL and MG-H1 free adducts were increased in patients with T1DM, increases in urinary excretion of FL often being less than expected due to metabolism by fructosamine 3-phosphokinase^16,22^. Since hyperglycemia and increased methylglyoxal have been evidenced as mediators of the development of diabetic nephropathy in experimental diabetes and linked to progression of clinical diabetic nephropathy^11,43–46^, it may be surprising that only urinary excretion of pentosidine free adduct was linked to early decline in GFR herein. Urinary excretion of glycation free adducts may be more closely associated with later stages of decline in GFR. Factors that may likely mask the association of urinary excretion of glycation free adducts to development of MA and early decline in GFR are: (i) high variability of the contribution to urinary excretion of major glycation free adducts from digested glycated proteins in food, producing increased dispersion of estimates of urinary excretion^47^; and (ii) urinary excretion of glycation free adducts vary from day-to-day whereas clinical development of MA and decline in GFR in patients with T1DM develops slowly over 10–20 year ^44,48^. Association of increased fractional excretion of glycation free adducts, as an in situ measure of renal function likely reflecting proximal tubular uptake and active secretion of these metabolites, may be a mechanistic biomarker that is little impacted by these factors.

The current clinical biomarker widely used to assess risk of early renal function decline is urinary albumin or ACR as reported herein. It is a predictor of decline in renal function but has limited sensitivity and specificity, with many patients with normoalbuminuria progressing to early renal function decline^49^. Clinical risk factors for decline in renal function in diabetic nephropathy include age, diabetes duration, HbA_1c_, systolic blood pressure, albuminuria, baseline GFR and retinopathy status. From recent studies, potential clinical biomarkers for early renal function decline in patients with T1DM and microalbuminuria are: plasma soluble tumor necrosis factor receptor isoforms 1 and 2 (TNFR1 and TNFR2) and E-selectin^50,51^, plasma kininogen and kininogen fragments^52^ and 7 metabolites identified in an unfocussed metabolomics study (C-glycosyltryptophan, pseudouridine, O-sulfotyrosine, N-acetylthreonine, N-acetylserine, N_6_-carbamoylthreonyl-adenosine, and N_6_-acetyllysine)^53^. Protein glycation, oxidation nitration adducts were not quantified in this metabolomics study. In a study assessing rapid development of diabetic nephropathy by measurment of glomerular basement membrane thickening in renal biopsies of patients with T1DM, increased plasma MG-HI, CEL, and CML free adducts were linked to rapid progression of diabetic nephropathy in a logistic regression model^11^. A further biomarker developed was a diagnostic algorithm based on the abundance of 273 urinary peptides (CKD273)^54^. The latter biomarker has been used to risk stratify patients in a clinical trial^55^. Useful clinical biomarkers for early renal function decline in diabetic nephropathy are those that are readily translated to the clinical chemistry laboratory, reliably and rapidly quantified, provide marked diagnostic improvement over current methods and are linked mechanistically to early-stage decline in renal function. With further validation, fractional excretion of one or more glycation adducts may meet these criteria.

The major limitations of this study are the relatively small size of the patient study groups and, for wider application, limited commercial availability of analytical standards^56^. In future studies it will be of interest to study changes in expression of amino acid transporters implicated in glycation adduct reuptake in renal proximal tubules in experimental models of diabetic kidney disease and in renal biopsies of clinical diabetic kidney disease. It is also of interest to explore association of genetic polymorphisms of amino acid transporters with early renal function decline in diabetic kidney disease.

## Conclusions

We found higher fractional excretion of 6 lysine and arginine-derived glycation adducts in patients with T1DM and MA with early decline in renal function, compared to patients with T1DM with and without MA and stable renal function. Higher fractional excretion of glycation free adducts may be an indicator of early functional decline of tubular cation transport protein function, known to be linked to chronic kidney disease, and may be a valuable biomarker for identification of patients with T1DM at risk of rapid decline in renal function.

## Methods

### Subject study groups and sampling

Subject group participants in this study were recruited and enrolled in the First Joslin Study of the Natural History of Microalbuminuria in Type 1 Diabetes, a follow-up cohort study of patients with T1DM new onset MA initiated in 1991 by the Internal Medicine and Pediatrics departments of the Joslin Diabetes Center (Boston, USA). Thereafter, members of the cohort returned to the clinic and random urine specimens were examined for MA. Details of the selection criteria and methods were published with the results of the screening study^57,58^. The protocol and consent procedures were approved by the Committee on Human Studies of the Joslin Diabetes Center. Written informed consent was obtained from all participants. The study registration number of the Joslin Diabetes Center Committee on Human Studies was: 88–17. The study was conducted in accordance with the Declaration of Helsinki on ethical principles for medical research.

From among the 267 patients who had persistent NA during the interval 1991 and 2004, we randomly selected 30 patients as a reference group with at least 12 years of follow-up as controls for this study. From the 109 patients who developed new-onset MA between 1992 and 1996, 86 patients had more than 10 years follow-up and we identified 55 of these for whom we had obtained urine and serum specimens during one of the biennial examinations carried out between 2000 and 2004. Measurement of urinary albumin-creatinine ratio (ACR) was used for both the diagnosis and follow-up of urinary albumin excretion (UAE) of this study. Urinary albumin concentration was measured by immune-nephelometry (Behring, Somerville, NJ, USA) and urinary creatinine concentration was measured by colorimetry (modified Jaffe reaction) on an Astra-7 automated system (Beckman Instruments, Brea, CA, USA)^57,58^. The patient’s level of UAE was defined according to a consensus of the last three measurements; a category of UAE was assigned if the level is confirmed by at least two out of three consecutive measurements, imposing time constraints on the intervals between measurements. The lower and upper limits of ACR for MA were: for men, 17 and < 250 µg/mg (1.9 and 28 mg/mmol); and for women, 25 and < 355 µg/mg (2.8 and 40 mg/mmol), respectively^59^. In order to detect persistent MA, measurements of ACR be separated by ≥ 8 weeks (median interval was 5 months). A fixed interval of two years was used to define the level of UAE over which the majority of patients (61%) had at least three ACR determinations.

Participants were also classified according to the presence of “early GFR decline”, determined using longitudinal measurements of glomerular filtration rate estimated by serum cystatin C (GFR_cystatin C_), and derived from the linear regression of log-transformed values for each individual over 10 or more years of follow-up. Early GFR decline was defined as percent change in GFR_cystatin C_ < -3.2%/year^44^. Twenty-two participants had early GFR decline and 33 had stable GFR. All 30 normoalbuminuric participants had stable GFR.

Baseline height and weight were abstracted from the medical records. BMI was calculated by the formula: weight (in kilograms) divided by height (in meters) squared.

### Measurement of protein glycation, oxidation and nitration adducts in urine

As described above, urine specimens were obtained within the first 4 years of MA onset (n = 55) and within the first 4 years of follow-up in those subjects with normoalbuminuria (n = 30)^14^. Urine ultrafiltrate was prepared passing an aliquot of urine (50 µl) through a 3 kDa microspin ultrafilter at 4 °C (10,000 g, 30 min). Urinary glycation, oxidation and nitration free adducts were determined by assay in the ultrafiltrate by stable isotopic dilution analysis liquid chromatography-tandem mass spectrometry (LC–MS/MS)—also called AGEomics; pentosidine was measured by concurrent fluorescence detection^15^. A detailed step-by-step protocol was published previously^60^, including description and discussion of the method and its strengths and weaknesses^56^. Briefly, an aliquot of urine ultrafiltrate (5 µl) was mixed with a cocktail of stable isotope-substituted internal standard analytes in initial mobile phase (0.1% trifluoracetic acid (TFA) in water), final volume 50 µl, and analysed by LC–MS/MS. For the chromatographic step, the mobile phase was 0.1% TFA in water with a custom multi-step gradient of increasing 0–50% acetonitrile; flow rate 0.2 ml/min. Elution was through two columns in series, with column switching to facilitate rapid elution of analytes of diverse hydrophobicity. The columns were: 5-*µ*m particle size HYPERCARB; column-1, 2.1 × 50 mm and column-2, 2.1 × 250 mm (Thermo Fisher Scientific, Loughborough, U.K.); column temperature was 30 °C. Eluate flow from 0–4 min was diverted to waste (containing non-volatile salts) and then switched to the mass spectrometer from 4–33 min for data collection. Analytes and stable isotope-substituted internal standards were detected by cationic electrospray ionisation (ESI) tandem mass spectrometry in multiple reaction monitoring (MRM) mode. The detection response is specific for chromatographic retention time and masses of molecular ion and fragment ion. Detection responses were optimised for molecular and fragment ion masses (± 0.1 Da), collision energy (± 1 eV) and capillary voltage (± 1 eV), as given previously^60^. Samples and calibration standards (6 known amounts of authentic standards covering the sample analyte content range mixed with stable isotopic standards) were analysed under identical conditions in the same run. The amount of analyte in the samples is deduced by interpolating the analyte/stable isotopic standard peak area ratio deduced from MRM chromatograms on calibration curves. Calibration curves were linear over the working analyte amount with R^2^ ≥ 0.993 for all analytes. Pentosidine was detected by in-line fluorescence detection; excitation/emission wavelengths 320/385 nm. Limits of detection of analytes and other analytic characteristics for analytes are given elsewhere^60^. The interbatch coefficient of variance of urinary analytes quantified herein was < 5%. In total, 12 analytes were measured: FL, CML, N_ε_-carboxyethyl-lysine (CEL), glyoxal-derived hydroimidazolone (G-H1), MG-H1, 3-deoxyglucosone-derived hydroimidazolones (3DG-H), N_ε_-carboxymethyl-arginine (CMA), glyoxal and methylglyoxal-derived lysine dimers, GOLD and MOLD, methionine sulfoxide (MetSO), N-formyl-kynurenine (NFK), dityrosine (DT) and 3-NT. Analyte concentrations were normalised to urinary creatinine and combined with previously determined serum concentrations to deduce fractional excretion values. Sample analysis was performed in 2007.

### Statistical analysis

Baseline clinical characteristics were compared using the Student’s t-test, the Wilcoxon rank-sum test, or the χ^2^-test, and 2 separate comparisons were made: 1) between those with NA (n = 30) and MA (n = 55) and 2) between those who demonstrated stable GFR (n = 63) and early GFR decline (n = 22)^14^. GFR measured longitudinally was determined using cystatin C and slopes were subsequently produced by the use of a linear model on log-transformed values to obtain percent change over time. To compare urinary excretion of glycation, oxidation and nitration free adducts, the Kruskal–Wallis test was applied for the 3 study groups and Mann–Whitney U test was applied to compare difference of medians of two study groups. To compare fractional excretion data, the Student’s t-test was performed on log-transformed values of the 12 free adducts. Correlation analysis was performed using non-parametric Spearman method. An α-level of 0.05 was used for tests of significance, and a Bonferroni correction was used for analyses of urinary and fractional excretion. Statistical analysis was performed using SAS version 9.1 (SAS Institute, Cary, NC, USA).

## Data Availability

The data used to support the findings of this study are available from the corresponding author upon request.

## References

[1] Koye DN, Magliano DJ, Nelson RG, Pavkov ME (2018). The Global Epidemiology of diabetes and kidney disease. Adv. Chronic Kidney Dis..

[2] International-Diabetes-Federation. *IDF Diabetes Atlas*. (Brussels, Belgium, 2017).

[3] Rossing P, de Zeeuw D (2011). Need for better diabetes treatment for improved renal outcome. Kidney Int..

[4] American Diabetes Association (2019). Standards of medical care in diabetes-2019. Diabetes Care.

[5] de Zeeuw D, Heerspink HJL (2016). Unmet need in diabetic nephropathy: failed drugs or trials?. Lancet Diabetes Endocrinol..

[6] Perkovic V, Jardine MJ, Neal B, Bompoint S, Heerspink HJL, Charytan DM, Edwards R, Agarwal R, Bakris G, Bull S, Cannon CP, Capuano G, Chu P-L, de Zeeuw D, Greene T, Levin A, Pollock C, Wheeler DC, Yavin Y, Zhang H, Zinman B, Meininger G, Brenner BM, Mahaffey KW (2019). Canagliflozin and renal outcomes in type 2 diabetes and nephropathy. New Engl. J. Med..

[7] Rabbani N, Thornalley PJ (2018). Advanced glycation end products in the pathogenesis of chronic kidney disease. Kidney Int..

[8] McCance DR, Dyer DG, Dunn JA, Baiue KE, Thorpe SR, Baynes JW, Lyons TJ (1993). Maillard reaction products and their relation to complications in insulin-dependent diabetes mellitus. J. Clin. Invest..

[9] Genuth S, Sun W, Cleary P, Gao X, Sell DR, Lachin J, Group DER, Monnier VM (2015). Skin advanced glycation endproducts (AGEs) glucosepane and methylglyoxal hydroimidazolone are independently associated with long-term microvascular complication progression of type i diabetes. Diabetes.

[10] Klein R, Horak K, Lee KE, Danforth L, Cruickshanks KJ, Tsai MY, Gangnon RE, Klein BEK (2017). The relationship of serum soluble receptor for advanced glycation end products (sRAGE) and carboxymethyl lysine (CML) to the incidence of diabetic nephropathy in persons with type 1 diabetes. Diabet. Care.

[11] Beisswenger PJ, Howell SK, Russell GB, Miller ME, Rich SS, Mauer M (2013). Early progression of diabetic nephropathy correlates with methylglyoxal-derived advanced glycation end products. Diabet. Care.

[12] Sagoo MK, Gnudi L (2018). Diabetic nephropathy: Is there a role for oxidative stress?. Free Rad. Biol. Med..

[13] Thornalley PJ, Rabbani N (2014). Detection of oxidized and glycated proteins in clinical samples using mass spectrometry–a user's perspective. Biochim. Biophys. Acta.

[14] Perkins BA, Rabbani N, Weston A, Ficociello LH, Adaikalakoteswari A, Niewczas M, Warram J, Krolewski AS, Thornalley P (2012). Serum levels of advanced glycation endproducts and other markers of protein damage in early diabetic nephropathy in type 1 diabetes. PLoS ONE.

[15] Thornalley PJ, Battah S, Ahmed N, Karachalias N, Agalou S, Babaei-Jadidi R, Dawnay A (2003). Quantitative screening of advanced glycation endproducts in cellular and extracellular proteins by tandem mass spectrometry. Biochem. J..

[16] Ahmed N, Babaei-Jadidi R, Howell SK, Beisswenger PJ, Thornalley PJ (2005). Degradation products of proteins damaged by glycation, oxidation and nitration in clinical type 1 diabetes. Diabetologia.

[17] Rabbani N, Thornalley PJ (2015). Dicarbonyl stress in cell and tissue dysfunction contributing to ageing and disease. Biochem. Biophys. Res. Commun..

[18] Delpierre G, Rider MH, Collard F, Stroobant V, Vanstapel F, Santos H, Van Schaftingen E (2000). Identification, cloning, and heterologous expression of a mammalian fructosamine-3-kinase. Diabetes.

[19] Swaminathan R, Major P, Snieder H, Spector T (2000). Serum creatinine and fat-free mass (lean body mass). Clin. Chem..

[20] James G, Sealey J, Alderman M, Ljungman S, Meuller F, Pecker M, Laragh JA (1988). Longitudinal study of urinary creatinine and creatinine clearance in normal subjects. Race, sex and age differences. Am. J. Hypertens..

[21] Karachalias N, Babaei-Jadidi R, Rabbani N, Thornalley PJ (2010). Increased protein damage in renal glomeruli, retina, nerve, plasma and urine and its prevention by thiamine and benfotiamine therapy in a rat model of diabetes. Diabetologia.

[22] Szwergold BS, Howell S, Beisswenger PJ (2001). Human fructosamine-3-kinase. Purification, sequencing, substrate specificity, and evidence of activity in vivo. Diabetes.

[23] Miyata T, Ueda Y, Horie K, Nangaku M, Tanaka S, de Strihou CVY, Kurokawa K (1998). Renal catabolism of advanced glycation end products: The fate of pentosidine. Kidney Internat..

[24] Grunwald S, Krause R, Bruch M, Henle T, Brandsch M (2006). Transepithelial flux of early and advanced glycation compounds across Caco-2 cell monolayers and their interaction with intestinal amino acid and peptide transport systems. Brit. J. Nutrit..

[25] Hellwig M, Geissler S, Matthes R, Peto A, Silow C, Brandsch M, Henle T (2011). Transport of free and peptide-bound glycated amino acids: synthesis, transepithelial flux at caco-2 cell monolayers, and interaction with apical membrane transport proteins. Chem. Bio. Chem.

[26] Anwar A, Abruzzo PM, Pasha S, Rajpoot K, Bolotta A, Ghezzo A, Marini M, Posar A, Visconti P, Thornalley PJ, Rabbani N (2018). Advanced glycation endproducts, dityrosine and arginine transporter dysfunction in autism - a source of biomarkers for clinical diagnosis. Mol. Autism.

[27] Fotiadis D, Kanai Y, Palacín M (2013). The SLC3 and SLC7 families of amino acid transporters. Mol. Aspects of Med.

[28] Makrides V, Camargo SMR, Verrey F (2014). Transport of amino acids in the kidney. Comprehens. Physiol..

[29] González M, Patel VB, Preedy VR, Rajendram R (2017). Regulation of expression and activity of L-arginine transporters by nutrients and hormones: a focus in transcriptional mechanisms regulated by glucose and insulin. L-Arginine in Clinical Nutrition.

[30] Chambers JC, Zhang W, Lord GM, van der Harst P (2010). Genetic loci influencing kidney function and chronic kidney disease. Nat. Genet..

[31] Chasman DI, Fuchsberger C, Pattaro C, Teumer A, Böger CA, Endlich K, Olden M, Chen M-H, Tin A, Taliun D, Li M, Gao X, Gorski M, Yang Q, Hundertmark C, Foster MC (2012). Integration of genome-wide association studies with biological knowledge identifies six novel genes related to kidney function. Hum Mol. Genet..

[32] Wang K, Kestenbaum B (2018). Proximal tubular secretory clearance - a neglected partner of kidney function. Clin. J. Am. Soc. Nephrol..

[33] Liu HC, Goldenberg A, Chen Y, Lun C, Wu W, Bush KT, Balac N, Rodriguez P, Abagyan R, Nigam SK (2016). Molecular properties of drugs interacting with SLC22 transporters OAT1, OAT3, OCT1, and OCT2: a machine-learning approach. J. Pharmacol. Exp. Ther..

[34] Moskovitz J, Weissbach H, Brot N (1996). Cloning and expression of a mammalian gene involved in the reduction of methionine sulfoxide residues in proteins. Proc. Natl. Acad. Sci. USA.

[35] Broer S (2008). Amino acid transport across mammalian intestinal and renal epithelia. Physiol. Revs..

[36] Wang F, Zhao Y, Niu Y, Wang C, Wang M, Li Y, Sun C (2012). Activated glucose-6-phosphate dehydrogenase is associated with insulin resistance by upregulating pentose and pentosidine in diet-induced obesity of rats. Horm. Metab. Res..

[37] Rabbani N, Xue M, Thornalley PJ (2016). Methylglyoxal-induced dicarbonyl stress in aging and disease: first steps towards glyoxalase 1-based treatments. Clin. Sci..

[38] Agalou S, Ahmed N, Babaei-Jadidi R, Dawnay A, Thornalley PJ (2005). Profound mishandling of protein glycation degradation products in uremia and dialysis. J. Am. Soc. Nephrol..

[39] Russo LM, Sandoval RM, Campos SB, Molitoris BA, Comper WD, Brown D (2009). Impaired tubular uptake explains albuminuria in early diabetic nephropathy. J. Am. Soc. Nephrol..

[40] McLellan AC, Thornalley PJ, Benn J, Sonksen PH (1994). The glyoxalase system in clinical diabetes mellitus and correlation with diabetic complications. Clin. Sci..

[41] Thornalley PJ, McLellan AC, Lo TWC, Benn J, Sonksen PH (1996). Negative association of red blood cell reduced glutathione with diabetic complications. Clin. Sci..

[42] Beisswenger PJ, Wood ME, Howell SK, Touchette AD, O'Dell RM, Szwergold BS (2001). α-Oxoaldehydes increase in the postprandial period and reflect the degree of hyperglycaemia. Diabet. Care.

[43] The Diabetes, C., Complications Trial/Epidemiology of Diabetes, I. & Complications Research, G. Retinopathy and Nephropathy in Patients with Type 1 Diabetes Four Years after a Trial of Intensive Therapy. *New Engl. J. Med.* **342**, 381–389 (2000). 10.1056/NEJM200002103420603 PMC2630213 10666428

[44] Perkins BA, Ficociello LH, Ostrander BE, Silva KH, Weinberg J, Warram JH, Krolewski AS (2007). Microalbuminuria and the risk for early progressive renal function decline in type 1 diabetes. J. Am. Soc. Nephrol..

[45] Giacco F, Du X, D’Agati VD, Milne R, Sui G, Geoffrion M, Brownlee M (2014). Knockdown of glyoxalase 1 mimics diabetic nephropathy in nondiabetic mice. Diabetes.

[46] Jensen TM, Vistisen D, Fleming T, Nawroth PP, Rossing P, Jørgensen ME, Lauritzen T, Sandbæk A, Witte DR (2016). Methylglyoxal is associated with changes in kidney function among individuals with screen-detected Type 2 diabetes mellitus. Diabet. Med..

[47] Xue M, Weickert MO, Qureshi S, Ngianga-Bakwin K, Anwar A, Waldron M, Shafie A, Messenger D, Fowler M, Jenkins G, Rabbani N, Thornalley PJ (2016). Improved glycemic control and vascular function in overweight and obese subjects by glyoxalase 1 inducer formulation. Diabetes.

[48] Perkins BA, Ficociello LH, Roshan B, Warram JH, Krolewski AS (2010). In patients with type 1 diabetes and new-onset microalbuminuria the development of advanced chronic kidney disease may not require progression to proteinuria. Kidney Int..

[49] Macisaac RJ, Jerums G (2011). Diabetic kidney disease with and without albuminuria. Curr. Opin. Nephrol. Hypertens..

[50] Gohda T, Niewczas MA, Ficociello LH, Walker WH, Skupien J, Rosetti F, Cullere X, Johnson AC, Crabtree G, Smiles AM, Mayadas TN, Warram JH, Krolewski AS (2012). Circulating TNF receptors 1 and 2 predict stage 3 CKD in type 1 diabetes. J. Am. Soc. Nephrol..

[51] Lopes-Virella MF, Baker NL, Hunt KJ, Cleary PA, Klein R, Virella G (2013). Baseline markers of inflammation are associated with progression to macroalbuminuria in type 1 diabetic subjects. Diabet. Care.

[52] Merchant ML, Niewczas MA, Ficociello LH, Lukenbill JA, Wilkey DW, Li M, Khundmiri SJ, Warram JH, Krolewski AS, Klein JB (2013). Plasma kininogen and kininogen fragments are biomarkers of progressive renal decline in type 1 diabetes. Kidney Int..

[53] Niewczas MA, Mathew AV, Croall S, Byun J, Major M, Sabisetti VS, Smiles A, Bonventre JV, Pennathur S, Krolewski AS (2017). Circulating modified metabolites and a risk of ESRD in patients with type 1 diabetes and chronic kidney disease. Diabet. Care.

[54] Pontillo C, Jacobs L, Staessen JA, Schanstra JP, Rossing P, Heerspink HJL, Siwy J, Mullen W, Vlahou A, Mischak H, Vanholder R, Zürbig P, Jankowski J (2017). A urinary proteome-based classifier for the early detection of decline in glomerular filtration. Nephrol. Dial Transplant..

[55] Lindhardt M, Persson F, Currie G, Pontillo C, Beige J, Delles C, von der Leyen H, Mischak H, Navis G, Noutsou M, Ortiz A, Ruggenenti PL, Rychlik I, Spasovski G, Rossing P (2016). Proteomic prediction and Renin angiotensin aldosterone system Inhibition prevention Of early diabetic nephRopathy in TYpe 2 diabetic patients with normoalbuminuria (PRIORITY): essential study design and rationale of a randomised clinical multicentre trial. BMJ Open.

[56] Rabbani N, Thornalley PJ (2020). Reading patterns of proteome damage by glycation, oxidation and nitration. Essays Biochem..

[57] Krolewski AS, Laffel LMB, Krolewski M, Quinn M, Warram JH (1995). Glycosylated hemoglobin and the risk of microalbuminuria in patients with insulin-dependent diabetes mellitus. New Engl. J. Med..

[58] Warram JH, Gearin G, Laffel L, Krolewski AS (1996). Effect of duration of type I diabetes on the prevalence of stages of diabetic nephropathy defined by urinary albumin/creatinine ratio. J. Am. Soc. Nephrol..

[59] Warram JH, Scott LJ, Hanna LS, Wantman M, Cohen SE, Laffel LM, Ryan L, Krolewski AS (2000). Progression of microalbuminuria to proteinuria in type 1 diabetes: nonlinear relationship with hyperglycemia. Diabetes.

[60] Rabbani N, Shaheen F, Anwar A, Masania J, Thornalley PJ (2014). Assay of methylglyoxal-derived protein and nucleotide AGEs. Biochem. Soc. Trans..

